# CpG ODN1826 as a Promising Mucin1-Maltose-Binding Protein Vaccine Adjuvant Induced DC Maturation and Enhanced Antitumor Immunity

**DOI:** 10.3390/ijms19030920

**Published:** 2018-03-20

**Authors:** Jing Jie, Yixin Zhang, Hongyue Zhou, Xiaoyu Zhai, Nannan Zhang, Hongyan Yuan, Weihua Ni, Guixiang Tai

**Affiliations:** Department of Immunology, College of Basic Medical Science, Jilin University, Xinjiang Street 125, Changchun 130021, China; jiejing15@mails.jlu.edu.cn (J.J.); zhangyx16@mails.jlu.edu.cn (Y.Z.); zhouhy16@mails.jlu.edu.cn (H.Z.); dixy15@mails.jlu.edu.cn (X.Z.); znn16@mails.jlu.edu.cn (N.Z.); yuanhy@jlu.edu.cn (H.Y.); niwh5566@jlu.edu.cn (W.N.)

**Keywords:** CpG ODN 1826, antitumor vaccine, MUC1-MBP, dendritic cells

## Abstract

Mucin 1 (*MUC1*), being an oncogene, is an attractive target in tumor immunotherapy. Maltose binding protein (MBP) is a potent built-in adjuvant to enhance protein immunogenicity. Thus, a recombinant MUC1 and MBP antitumor vaccine (M-M) was constructed in our laboratory. To enhance the antitumor immune activity of M-M, CpG oligodeoxynucleotides 1826 (CpG 1826), a toll-like receptor-9 agonist, was examined in this study as an adjuvant. The combination of M-M and CpG 1826 significantly inhibited *MUC1*-expressing B16 cell growth and prolonged the survival of tumor-bearing mice. It induced MUC1-specific antibodies and Th1 immune responses, as well as the Cytotoxic T Lymphocytes (CTL) cytotoxicity in vivo. Further studies showed that it promoted the maturation and activation of the dendritic cell (DC) and skewed towards Th1 phenotype in vitro. Thus, our study revealed that CpG 1826 is an efficient adjuvant, laying a foundation for further M-M clinical research.

## 1. Introduction

Mucin 1 (MUC1) is a tumor-associated antigen that is expressed on the apical surface of epithelial cells. In cancers, such as lung, breast, ovarian, prostate, and pancreatic cancer, as well as in several malignant hematological tumors, it is aberrantly overexpressed. Different modes of expression between normal and aggressive forms make them ideal targets for immunotherapy [1,2]. Peptide-based MUC1 vaccines, antitumor-associated MUC1 monoclonal antibodies, adoptive transfer of MUC1-specific cytotoxic T lymphocytes (CTLs), and recombinant poxvirus vector vaccines have undergone numerous pre-clinical and clinical trials that target the tumor-associated antigen MUC1 in recent years [3]. Currently, most MUC1-based vaccines are in phase I and II clinical trials [4], only a small number entered Phase IIB or III trials, such as Tecemotide and TG4010 [5].

Adjuvants are required to make the protein a highly efficient vaccine, because protein-based vaccines are usually poorly immunogenic [6]. Maltose-binding Protein (MBP) is used as a chaperone in various experimental vaccines in which recombinant protein-MBP has been shown to increase immunogenicity [7]. Dendritic cells (DCs) maturation is promoted by MBP through the signaling of Toll-like receptor (TLR)-4 [8]. Our previous experiments indicated that MBP directly induced NK and Th1 activation and macrophage M1 polarization via TLR2 [9,10,11]. Moreover, MBP combined with Bacillus Calmette-Guérin (BCG), induced synergistic Th1 activation via TLR2, TLR4, and TLR9 pathways [12]. Thus, a recombinant MUC1-MBP protein vaccine (M-M) consisting of the human gene *MUC1* and MBP was constructed by inserting seven tandem repeats encoding the human *MUC1* gene into the pMAL-p2 expression vector. BCG is a live, attenuated strain of mycobacterium bovis, which is an effective adjuvant in cancer immunotherapy and activates the TLR2/TLR9 signaling pathway [13]. In previous studies, M-M combined with BCG induced the Th1 dominant immune response, NK cell activity, MUC1-specific cytotoxic T lymphocyte (CTL) killing activity, and mouse *MUC1* expression B16 cell (B16-*MUC1*) growth inhibition, but induced arthritis or local nodules in rats and cynomolgus monkeys [14,15]. BCG causes severe local and systemic side effects, even though it is the most effective treatment for high-risk noninvasive bladder cancer [16]. In addition, the stability problems associated with bacterial vaccines are difficult to control [17]. Therefore, it is difficult to control in drug development. These problems with the application of BCG motivated us to identify an alternative adjuvant that is considerably safer and more effective.

The objective of most cancer vaccines is to generate a vast amount of tumor-specific CTL. CTL is believed to play a central role in tumor eradication because cellular, rather than humoral, immunity is superior in cancer immunotherapy [18]. TLRs are a class of pattern recognition receptors that play an important role in the activation of innate immunity [19]. Various TLR agonists are currently undergoing clinical trials for their effectiveness to orchestrate antitumor immunity. CpG oligodeoxynucleotides (CpG) mimic the immunostimulatory activity of bacterial DNA and TLR9 signaling. CpG is a chemically-synthesized compound and its quality control is superior to that of BCG, though Heikenwalder found CpG exerts toxicity, such as lymphoid morphology and functionality alternation, multifocal liver necrosis and hemorrhagic ascites in previous research when injected four times at days 2, 7, 14, and 20 with the dose of 60 μg/mouse [20]. CpG is a kind of vaccine adjuvant that promotes Th1 immune responses and enhances the immunogenicity of many vaccines with high safety in some clinical research [21,22,23,24]. The reasonable dose and immunization schedule are important for optimal antitumor effect. A-type (CpG 1585) and B-type (CpG 1826) CpG are well known. The A-type is especially potent in activating human plasmacytoid dendritic cells (pDCs) produce high amounts of IFN-α and induce lymph node cDCs to mature, whereas the B-type more importantly stimulates strong B cell, NK cell, and pDCs activation; therefore, it is a very potent Th1 adjuvant and has anti-tumor activity [25]. The human clinical adjuvant activity trials of TLR9 ligands have focused on B-type CpG [26,27].

In this study, we compared the antitumor activity elicited by various adjuvants, primarily focusing on the TLR agonists, by detecting the immunological activity and establishing transplantation tumor models. We also explored the possible mechanism and investigated if there is a promotion of DC activation when M-M is combined with CpG 1826 (M-M + CpG 1826). Our study revealed that the anti-tumor effects of M-M combined with CpG 1826 was better than BCG, laying a foundation for further clinical research using M-M anti-tumor vaccine.

## 2. Results

### 2.1. Combination of M-M and CpG 1826 Inhibited B16-MUC1 Growth in Mice

**Figure 1:**
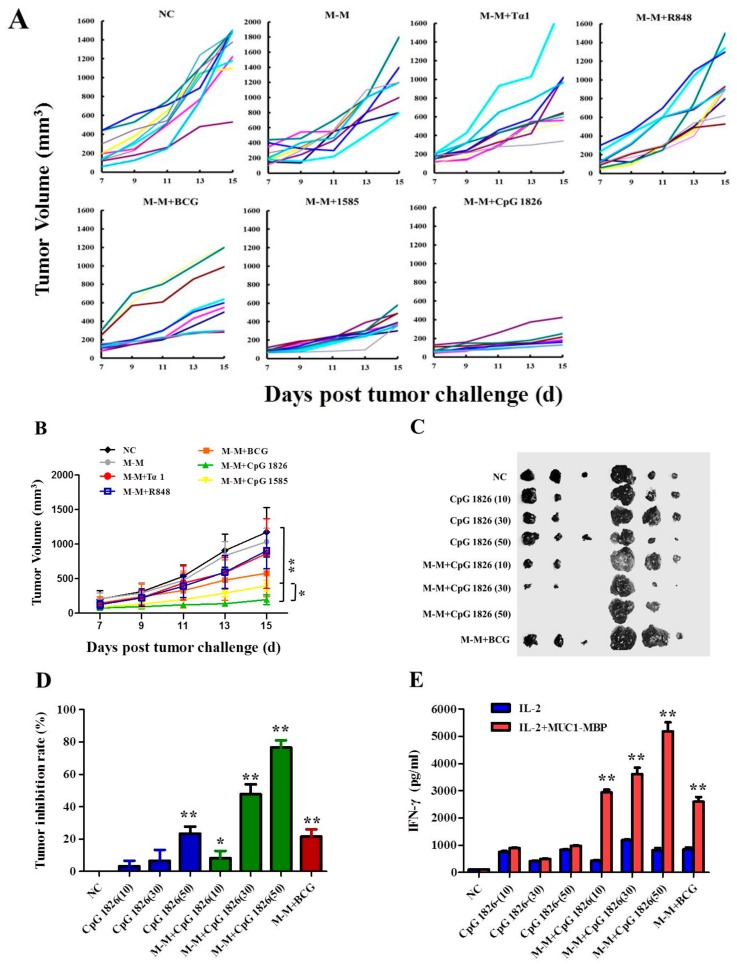
Anti-tumor effect of different adjuvants combined with M-M. (**A**) The anti-tumor effect of different adjuvants combined with M-M. Mice were divided into seven groups, and each group (*n* = 10) were immunized as followings: M-M, M-M + Tα1, M-M + R848, M-M + BCG, M-M + CpG 1585, M-M + CpG 1826, or PBS on day –21 and –7 and were then subcutaneous injected (s.c.) with 2 × 10^6^ B16-*MUC1* melanoma cells on day 0. Each line represents the tumor growth kinetics in each mouse. (**B**) The mean tumor growth curves are given by tumor volume. (**C**) The dose effect of CpG 1826 on the growth of B16-*MUC1* melanoma. Eight group of mice (*n* = 6) were immunized two times with CpG 1826 10, 30, and 50 μg alone or in combination with M-M (50 μg). M-M + BCG represent M-M combined BCG. The mice were sacrificed on day 24 after tumor inoculation (5 × 10^5^ B16-*MUC1* melanoma cells). (**D**) The tumor inhibition rate. Tumor inhibition rate (%) = (1 − experimental group total tumor weight/control group) × 100%. (**E**) Splenocytes obtained from the immunized mice with different dose of CpG 1826. The production of IFN-γ was detected in splenocytes supernatants stimulated by IL-2 or IL-2 + MUC1-MBP. Six mice per group were analyzed. * *p* < 0.05, ** *p* < 0.01 vs. the negative control (NC) group.

To obtain an improved antitumor effect of M-M, a series of adjuvants were applied in the present study. The mice were immunized with M-M combined with PBS, CpG 1826, Tα1, BCG, CpG 1585, or R848. Mice were immunized two times. One week after the final immunization, a tumor challenge was performed. The results showed that the tumor growth rate in the immunized groups was slower than that of the PBS group. Specifically, the M-M + CpG 1826 group showed the smallest tumor volume (Figure 1A,B). In order to study the antitumor effect of CpG 1826 combined with M-M, different doses of CpG 1826 were investigated. The results showed that the adjuvant alone group is not as effective as the combination group in tumor inhibition rate, respectively. M-M combined with CpG 1826 had a potent dose-dependent antitumor activity (Figure 1C,D). The combination of CpG 1826 at 50 μg drastically reduced the tumor growth. At the same time, the highest amount of IFN-γ was detected in the CpG 1826 50 μg group (Figure 1E). All of these findings indicated that CpG 1826 was a good adjuvant for the antitumor vaccine M-M.

### 2.2. M-M and CpG 1826 Enhance the Antitumor Response by Inducing MUC1-Specific Humoral and Cellular Immune Responses

**Figure 2:**
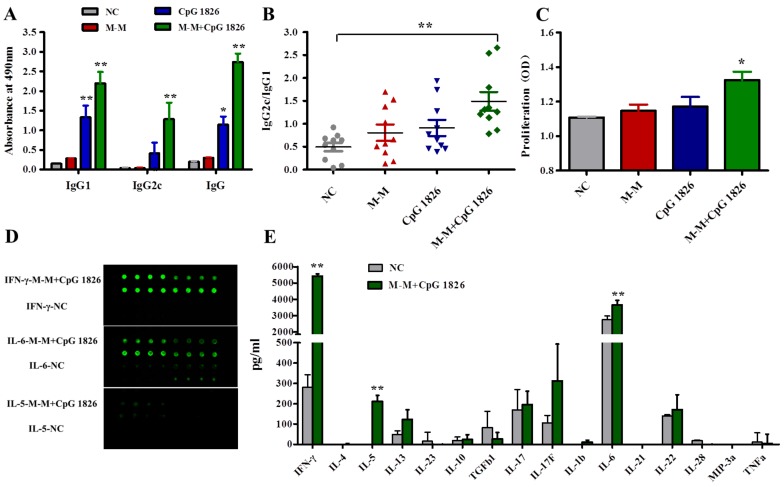

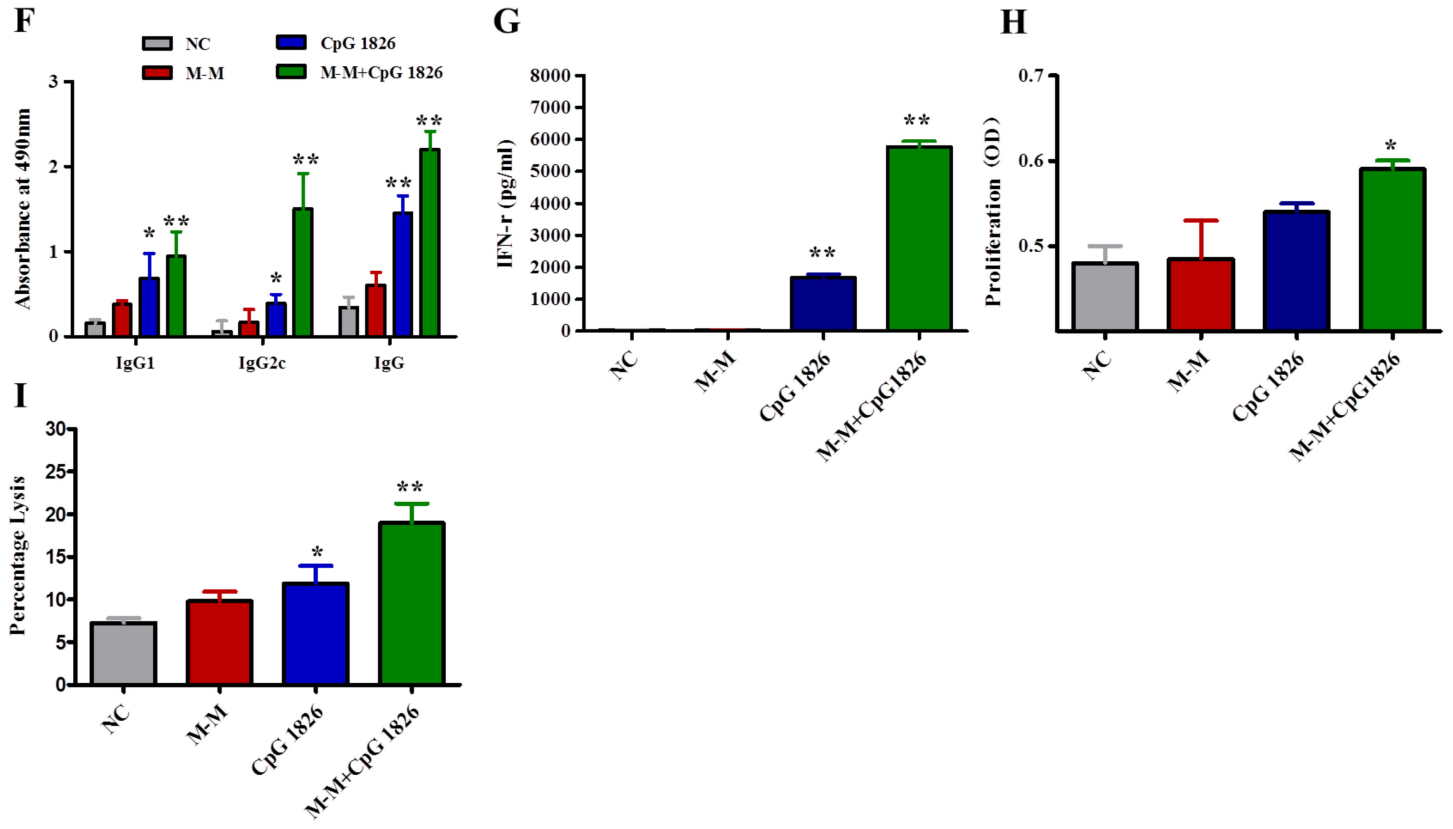
M-M combined with CpG 1826 synergistically enhances the anti-tumor response by inducing MUC1-specific humoral and cellular immune responses. (**A**–**E**) Four groups of mice (*n* = 10) were injected s.c. with PBS, M-M, CpG 1826, or M-M + CpG 1826 on day –21 and –7. On day 0, the sera were collected for the MUC1-specific antibody assay. The splenic mononuclear cells from each group were stimulated in vitro with a specific MUC1 peptide (20 μg/mL) for five days, and then a cell proliferation assay and cytokine assay were carried out. (**A**) MUC1-specific IgG, IgG1, and IgG2c levels in the sera of the immunized mice were determined by ELISA on day 7 after the last immunization. (**B**) Serum IgG2c/IgG1 ratio. The data represent the mean of ten mice per group. (**C**) The lymphocyte proliferation of the different immunized mice was detected by the WST-1 assay. (**D**) The original image of the chip analysis of the splenocyte cytokine secretion by the Quantibody^®^ array. Each mouse was replicated four times, and each group consisted of four mice. (**E**) The cytokine secretion detected by Quantibody^®^ array is expressed as the mean ± standard deviation and is shown in a bar graph. Th1, IFN-γ secreting cells; Th2, IL-4, IL-5, IL-6 IL-13, IL-23 secreting cells; Treg, IL-10, TGF-b1; Th17, IL-17, IL-17F secreting cells. The data represent the mean of four mice per group. * *p* < 0.05, ** *p* < 0.01 vs. NC group. (**F**–**H**) Four group of mice (*n* = 10) were injected s.c. with PBS, M-M, CpG 1826 or M-M + CpG 1826 on day –21 and –7. On day 0, a tumor challenge was performed with subcutaneous injection of 5 × 10^5^ B16-*MUC1* cells. On day 14, the sera were collected for the MUC1-specific antibody assay. The splenic mononuclear cells from each group were stimulated in vitro with a specific MUC1 peptide (20 μg/mL) for five days, and then a cell proliferation assay, cytokine assay were carried out. (**F**) MUC1-specific IgG, IgG1 and IgG2c levels in the sera of the immunized mice were determined. (**G**) The IFN-γ secretion is detected by ELISA (**H**) lymphocyte proliferation of the different immunized mice was detected by the WST-1 assay. (**I**) Effects of M-M combined with CpG 1826 on MUC1-specific CTL killing activity. Four groups of mice (*n* = 10) were injected s.c. with PBS, M-M, CpG 1826, or M-M + CpG 1826 on day –21 and –7. On day 0 splenic mononuclear cells were isolated. The splenic mononuclear cells from each group were stimulated in vitro with a specific MUC1 peptide (20 μg/mL) for five days, and then CTL cytotoxicity assay was carried out. Statistical significance compared with other groups was represented as follows: * *p* < 0.05, ** *p* < 0.01 vs. the NC group.

To investigate the antitumor mechanism of M-M combined with CpG 1826, a MUC1 specific antibody titer and antibody subtypes were assessed by testing the sera from the immunized mice on the 7th day after the last immunization. Mice immunized with M-M + CpG 1826 developed the highest levels of anti-MUC1 IgG, IgG1, and IgG2c antibody titers compared with other groups (Figure 2A). This finding implied that M-M + CpG 1826 induced a MUC1-specific humoral immune response in mice. The ratio of IgG2c/IgG1 was highest in the mice immunized with M-M + CpG 1826 (Figure 2B), indicating a strong Th1 response. More importantly, M-M + CpG 1826 induced MUC1-specific splenocyte proliferation compared with the other groups (Figure 2C), suggesting that splenocyte proliferation was antigen-dependent. IFN-γ production, as measured by the Quantibody array, was significantly higher in cells from mice vaccinated with M-M + CpG 1826 relative to cells from mice in the NC group. IL-5 and IL-6 were moderately increased, which represents the Th2 response (Figure 2D). At the same time, M-M + CpG 1826 had no significant effects on the Treg or Th17 cell subtypes, which are characterized by TGF-β1, IL-10, and IL-17 (Figure 2E). Taken together, Th1/Th2 cytokines may be implicated in the M-M + CpG 1826 antitumor mechanism, especially the Th1 cytokines.

After tumor challenge, mice immunized with M-M + CpG 1826 developed the highest levels of sera anti-MUC1 antibody titers compared with other groups (Figure 2F). Compared with the NC group, the M-M + CpG 1826 vaccination significantly increased the production of IFN-γ (Figure 2G). More importantly, M-M + CpG 1826 induced MUC1-specific splenocytes proliferation compared with the other groups (Figure 2H). It can be seen that M-M + CpG 1826 also produces antitumor immune response.

CTL killing activity is a gold standard measurement used to determine the efficacy of tumor vaccine. To detect whether M-M + CpG 1826 immunization is capable of inducing MUC1-specific CTL activity in mice, a B16 mouse melanoma cell line stably expressing human *MUC1* was established as target cells. Highest cytotoxicity was observed at (E): T ratio of 25:1 against B16-*MUC1* cells. Splenocytes from mice immunized with CpG 1826 alone could also lyse cells compared to the NC group, although at a lower cytotoxicity than the M-M + CpG 1826-immunized group (Figure 2I). These findings indicate that M-M + CpG 1826 immunization induced stronger MUC1-specific CTL activity compared with CpG 1826 alone.

### 2.3. M-M Combined with CpG 1826 Promote DC Maturation In Vivo and In Vitro

**Figure 3:**
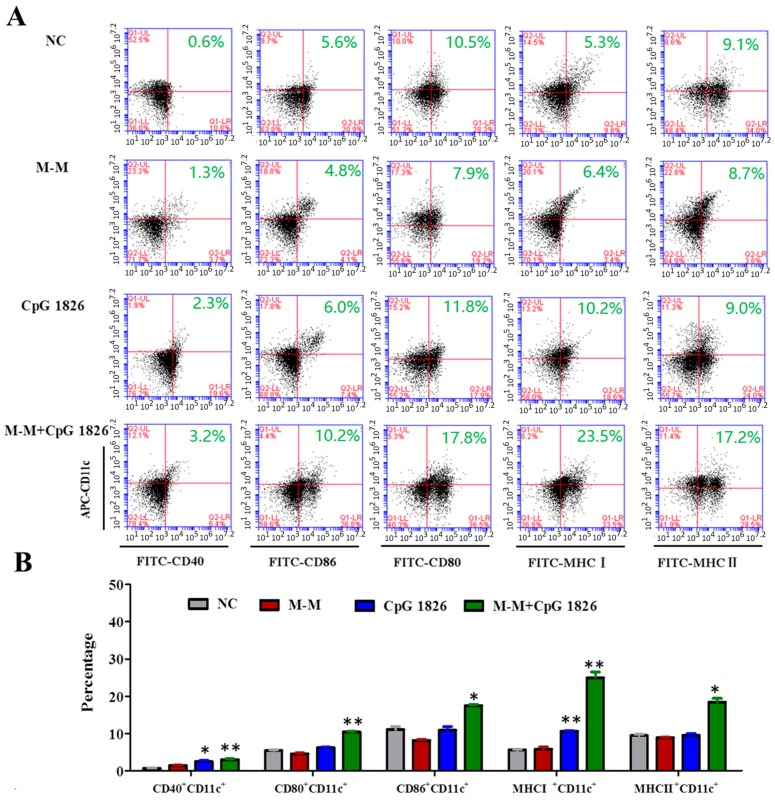

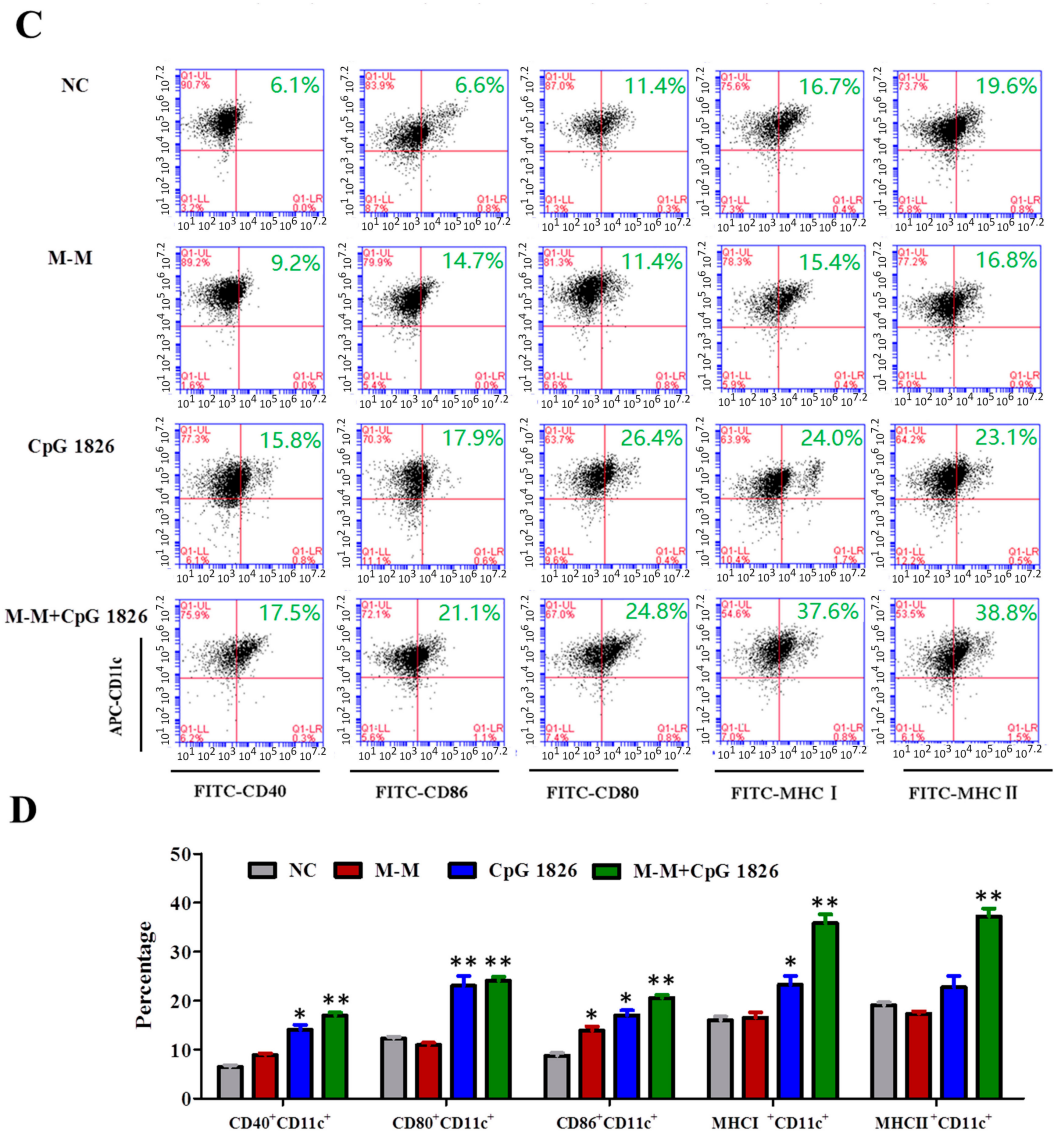
Effects of M-M, CpG 1826 on dendritic cell (DC) maturation in vivo and in vitro. (**A**,**B**) In vivo study. The draining lymph node was isolated on day 0 after an s.c. injection of PBS, M-M, CpG 1826 or M-M + CpG 1826 in the flank of the C57BL/6 mice on day –21 and –7. The draining lymph node was made into single cell suspensions. (**A**) The expression of major DCs surface markers was analyzed by flow cytometry. (**B**) The in vivo percentage of the (double positive) DC cells is expressed as the mean ± standard deviation and is shown in a bar graph. (**C**,**D**) In vitro study. (**C**) The percentage of dual-positive (DP) cells is shown in the flow cytometry histogram. The BMDCs were analyzed for the expression of CD40^+^CD11C^+^, CD80^+^CD11C^+^, CD86^+^CD11C^+^, MHCI^+^CD11C^+^, MHCII^+^CD11C^+^ by flow cytometry after stimulation with PBS, M-M, CpG 1826, or M-M + CpG 1826 for 48 h in vitro. (**D**) The in vitro percentage of (DP) cells is expressed as the mean ± standard deviation and is shown in a bar graph. The data are representative of three independent experiments. * *p* < 0.05, ** *p* < 0.01 vs. the NC group.

DC maturation is associated with a wide range of cellular changes, such as the increased expression of surface MHC class I and II molecules and co-stimulatory molecules [28]. To assess the impact of M-M + CpG 1826 on DC maturation, the expression levels of CD11c^+^, CD40^+^, CD80^+^, CD86^+^, MHCI^+^, and MHCII^+^ on the DCs within the dLNs were analyzed by flow cytometry. The expressions of CD40^+^CD11c^+^, CD80^+^CD11c^+^, CD86^+^CD11c^+^, MHCI^+^CD11c^+^, and MHCII^+^CD11c^+^ in the M-M + CpG 1826 group were significantly upregulated compared to the NC group (Figure 3A,B), while CpG 1826 alone slightly increased the percentage of CD40^+^CD11c^+^, and MHCI^+^CD11c^+^, indicating a stronger effect on DC maturation of the M-M + CpG 1826 in dLNs. In vitro study, the combination of M-M and CpG 1826 strongly increased the percentage of CD40^+^CD11c^+^, CD80^+^CD11c^+^, CD86^+^CD11c^+^, MHCI^+^CD11c^+^, and MHCII^+^CD11c^+^ (*p <* 0.01), while M-M alone slightly increased the percentage of CD86^+^CD11c^+^ (*p <* 0.05), CpG 1826 alone slightly increased the percentage of CD40^+^CD11c^+^, CD86^+^CD11c^+^, MHCI^+^CD11c^+^ (*p* < 0.05), and CD80^+^CD11c^+^ (*p* < 0.01), (Figure 3C,D). All of these findings demonstrate M-M combined with CpG 1826 induced the stronger maturation of DC.

### 2.4. Combination of M-M and CpG 1826 Promotes the Th1 Polarization of CD4^+^ T Cell Co-Cultured with BMDCs

**Figure 4:**
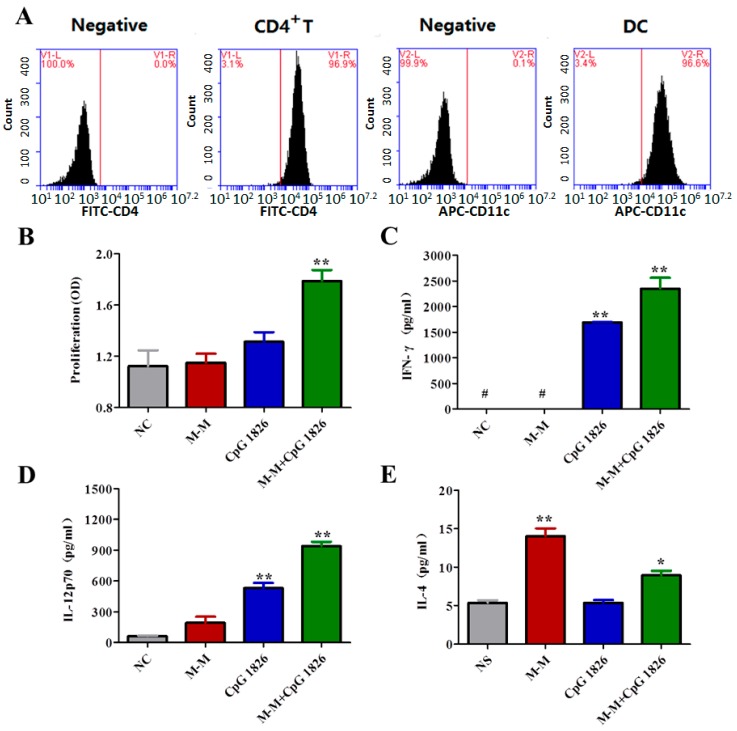
CD4^+^ T cell activation is enhanced by a co-culture with BMDCs stimulated with the combination of M-M and CpG 1826 in vitro. (**A**) The isolation of CD4^+^ T cells from the spleen samples from immunized mice and BMDCs from untreated mice. The CD4^+^ T cell and DC percentage was analyzed by flow cytometry. The purity of the CD4^+^ T cells was 96.9%. The purity of the DCs was 96.6%. (**B**) M-M and CpG 1826 synergistically increased the proliferation of co-cultured CD4^+^ T cells and DCs. (**C**–**E**) The production of IFN-γ, IL-12p70, and IL-4 in the CD4^+^ T cells cocultured with DCs, as detected by ELISA. The CD4 T cells were co-cultured with the DCs at a ratio of 50:1. All the experiments were repeated three times, and all the data are expressed as the mean ± SD (*n* = 3). # represents production <25 pg/mL. * *p* < 0.05, ** *p* < 0.01 vs. NC group.

To investigate whether BMDC treated with the combination of M-M and CpG 1826 affect the CD4^+^ T cell polarization, CD4^+^ T cells and BMDC cells were sorted. The purity of sorted CD4^+^ T cells and BMDC cells used in the present study was 97.6% and 96.6%, respectively (Figure 4A). As shown in Figure 4B, M-M + CpG 1826 significantly increased the proliferation of co-cultured CD4^+^ T cells and BMDC compared with NC group. To study the effect of the combination of M-M and CpG 1826 on the activation of CD4^+^ T cells co-cultured with BMDCs, the secretion of IFN-γ, IL-4, and IL-12p70 were examined. Co-culturing CD4^+^ T cells with BMDCs stimulated with M-M and CpG 1826 significantly increased the production of IFN-γ, IL-12p70, and IL-4 compared NC groups, suggesting the maturation and activation of the DCs and the polarization of the CD4^+^ T cells towards the Th1 phenotype (Figure 4C,E).

### 2.5. Combination of M-M and CpG 1826 Enhanced the Prophylactic and Therapeutic Antitumor Immune Activity

**Figure 5:**
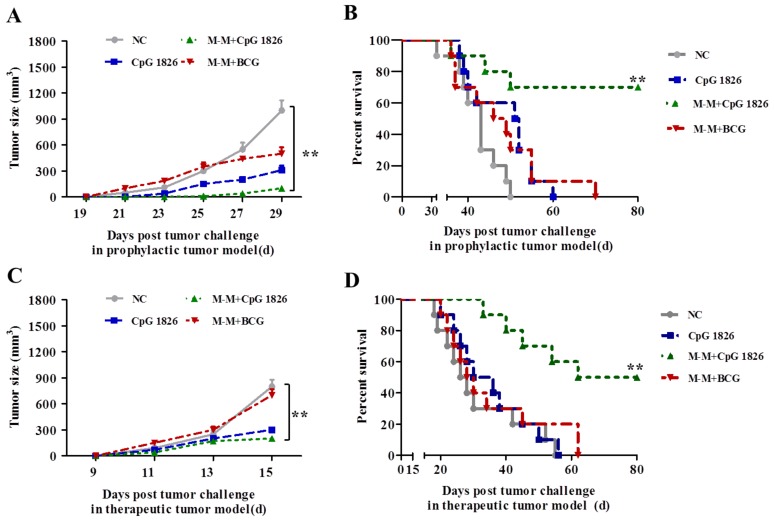
Role of M-M + CpG 1826 in prophylactic and therapeutic tumor models. (**A**–**B**) M-M + CpG 1826 vaccine induced a protective effect in a prophylactic tumor model. Four groups of mice (*n* = 10) were injected s.c. with PBS, CpG 1826, M-M + CpG 1826, or M-M + BCG on day –21 and –7 and were then inoculated s.c. with 5 × 10^5^ B16-*MUC1* melanoma cells on day 0. Tumor volume was measured every two days, and the survival of the mice was calculated. The PBS-injected mice were used as a negative control. ** *p* < 0.01 vs. NC group. (**A**) The mean tumor growth curves given by the tumor volume. Each line represents the mean tumor growth kinetics of ten mice in each group. (**B**) Survival time of the mice. (**C**,**D**) The M-M + CpG 1826 vaccine confers therapeutic protection against melanoma. Four groups of mice (*n* = 10) were inoculated with 5 × 10^5^ B16-*MUC1* melanoma cells on day 0 and were then injected s.c. with PBS, CpG 1826, M-M + CpG 1826 or M-M + BCG on days 7 and 21. (**C**) The mean tumor growth curves given by tumor volume. (**D**) Survival time of the mice.

To study the tumor protective role of M-M + CpG 1826, the prophylactic and therapeutic models were constructed by injecting mice subcutaneously with B16-*MUC1* tumor cells. In the prophylactic model, tumor growth was significantly slower in the mice vaccinated with M-M + CpG 1826 compared with NC or M-M + BCG (Figure 5A). At 50 days after the tumor challenge, the survival rate in the NC, CpG 1826, M-M + BCG, and M-M + CpG 1826 groups were 0%, 60%, 30%, and 70%, respectively. At 80 days after the tumor challenge, all of the other groups were dead, while the M-M + CpG 1826 group survival rate was 70% (Figure 5B). Taken together, these results suggest that CpG 1826 combined with M-M significantly inhibited the tumor growth and prolonged survival, while the individual CpG 1826 did not elicit a high protective immunity in the prophylactic model.

Additionally, in the therapeutic model, there was a significant delay in tumor growth when the M-M + CpG 1826-immunized group were compared with the M-M + BCG-immunized group (Figure 5C). At 55 days after the tumor challenge, the survival rate in the NC, CpG 1826, M-M + BCG, and M-M + CpG 1826 groups were 0%, 0%, 20%, and 60%, respectively. At 80 days after the tumor, all of the other groups were dead, while the M-M + CpG 1826 group survival rate was 50% (Figure 5D). These results suggested that immunization with M-M + CpG 1826 is capable of protecting mice against a lethal challenge with B16-*MUC1* both in prophylactic and therapeutic models.

## 3. Discussion

Our previous study showed that the M-M + BCG anti-tumor vaccine significantly inhibited B16-*MUC1* cell growth in mice, but induced arthritis or local nodules in rats and cynomolgus monkeys in pre-clinical toxicity evaluation. The side effects of the application of BCG drove us to identify an alternative adjuvant for the antitumor vaccine M-M, which is considerably safer and more effective. In this study, TLR9 agonists (CpG 1585, CpG 1826, CpG 2006), TLR7/8 agonists (R848), Tα1 and BCG were selected as the adjuvant candidates. M-M combined with CpG 1826 displayed the more prominent antitumor effect compared with others.

To explore the possible mechanism, an immune activity study was performed using sera, spleen and dLNs of immunized mice. More importantly, after tumor challenge the antibodies IgG2c and IFN-γ produced by M-M + CpG 1826 group were higher than that of CpG 1826 alone group. It can be seen that although M-M + CpG 1826 also produces a humoral response during tumor resistance, the cellular immune response plays a major role, suggesting the promising potential applications of M-M + CpG 1826 in the treatment of cancer. It appears that a Th/Treg cell imbalance in mice causes ineffective anti-MUC1 responses [29]. The mice immunized with M-M + CpG 1826 did not exhibit an increased frequency of the Treg subtype. The vaccine-induced MUC1-specific Tregs are balanced by an efficient MUC1-specific Th cell response.

The lack of effective MUC1-specific T cells in cancer patients is considered a major obstacle in generating an effective antitumor immunity [30]. Th cells are needed to work together with CD8^+^ T cells to establish an effective CTL memory. CTL killing activity is a gold standard of measurements to determine the effectiveness of a tumor vaccine. We selected three kinds of target cells: B16-neo, LLC1, and B16-*MUC1*. B16-neo does not express murine *MUC1*, whereas LLC1 expresses murine *MUC1*. Due to the low *MUC1* homology between humans and mice, we constructed B16-*MUC1* that expressed human *MUC1* as a specific target cell for this study. In the experiment, there was no significant difference of CTL cytotoxicity against LLC, B16-neo, or NK cytotoxicity against YAC-1, but with significant difference of CTL cytotoxicity against B16-*MUC1* cells. The results indicate that the vaccine could produce specific killing against human *MUC1*.

It is well-known that DCs are a bridge that links the innate and adaptive immunity [31]. Therefore, the maturation of DCs in draining lymph nodes in immunized mice and in vitro was analyzed. We found a synergistic enhancement of the M-M and CpG 1826 on DC maturation. The upregulation of surface costimulatory molecules on DC is essential for antigen presentation and T cell development [32]. M-M + CpG 1826 activated DCs displayed a professional APC function in an allogeneic mixed lymphocyte reaction. These results indicated that the combination of M-M + CpG 1826 induced the maturation of DCs, indirectly promoted the proliferation of co-cultured BMDC and CD4^+^ T cells, and induced CD4^+^ T cells skewed towards a MUC1-specific Th1 phenotype. B-type CpG 1826 stimulates strong B cell, NK cell, as well as pDCs activation. The IFN-α, produced by pDCs, directly activate CD8^+^ T cells, as well as BMDC maturation which, in turn, indirectly activated T cells [33,34]. Consistent with our findings, there is a similar maturation of BMDCs in murine and melanoma patient lymph nodes [35,36].

Next, the protective and therapeutic effects of M-M + CpG 1826 on tumor growth were also investigated in the study. Compared with other vaccines, the work described in the present report showed that the overall increase of the animal survival rate was most notably observed in the therapeutic model [37,38]. At 80 days after a tumor challenge, M-M + CpG 1826 exhibited a prominent 50% survival rate. It is well-known that in the B16 murine melanoma model, vaccination strategies that are efficacious in the prophylactic model, whereas it often fails in the therapeutic model. This further reflects the superiority of CpG 1826 as a very promising M-M vaccine adjuvant to enhance antitumor immunity.

**Figure 6:**
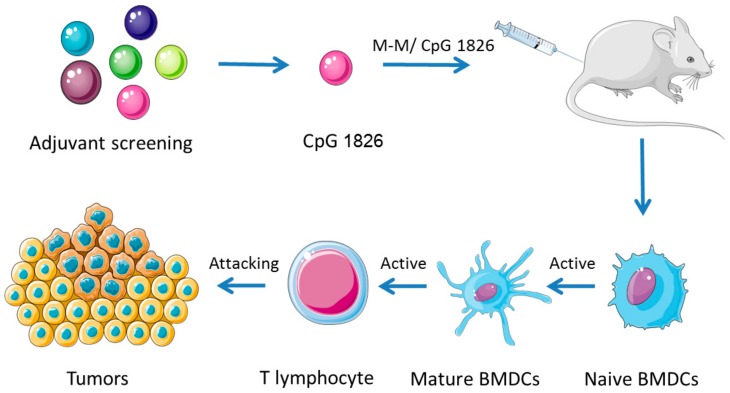
The schematic outline of CpG 1826 as a promising M-M vaccine adjuvant induced DC maturation and enhanced antitumor immunity. CpG 1826 displayed the more prominent effect compared with a series adjuvants. Through in vitro and in vivo study, we found the combination of M-M and CpG 1826 strongly enhanced the maturation of DC and, thus, activated T lymphocytes. Collaboratively, better prophylactic and therapeutic tumor immunotherapy effect were obtained.

In conclusion, we observed that the combination of M-M and CpG 1826 strongly enhanced the maturation of DC and, thus, activated T lymphocytes, and displayed the more prominent antitumor effect compared with a series adjuvants (Figure 6). In clinical trials, B-type CpG has received considerable interest as an adjuvant for cancer vaccines [39]. However, single CpG only has modest antitumor activity [40]. Thus, the combination of adjuvant and tumor antigen is critical. *MUC1* is an ideal target for immunotherapy. Using human MUC1 in a mouse model did have some limitations, such as safety and effectiveness. We have to admit that this experiment only shows that the vaccine used in mouse models produced specific killing of human MUC1, with the role of inhibiting tumor growth. These studies lay a foundation for further research on M-M anti-tumor vaccines, but more systematic and comprehensive clinical evaluation is needed.

## 4. Materials and Methods

### 4.1. Cell Lines

Murine B16 melanoma cells were transfected with the pcDNA3-*MUC1* VNTR plasmid encoding for human *MUC1* VNTR peptide. The cell line was selected in complete medium containing G418 (600 mg/L) (Sigma-Aldrich, St. Louis, MO, USA) for MUC1^+^ cell clones, and the stable monoclonal transfected cells were verified by fluorescence microscopy. B16-*MUC1* cells were used to establish a tumor model. Complete medium was prepared with Iscove’s Modified Dulbecco’s Medium (IMDM) (Gibco-BRL, Carlsbad, CA, USA) supplemented with 10% fetal bovine serum (FBS, Invitrogen, Carlsbad, CA, USA) and antibiotics (100 U/mL penicillin, 100 U/mL streptomycin) for the culturing of B16-*MUC1* cells [9].

### 4.2. Immunization

C57BL/6 mice (6–8 weeks) were purchased from the HFK Bioscience.co (Beijing, China) and maintained under specific pathogen-free conditions. The experimental manipulation of mice was conducted in accordance with the National Institute of Health Guide for the Care and Use of Laboratory Animals and the approval of the Scientific Investigation Board of Science and Technology of Jilin Province (Changchun, China). In addition to adjuvant screening experiments, in another experiment each mouse was immunized with CpG 1826 (50 μg), or M-M (50 μg) in the presence of CpG 1826, or not, subcutaneously in the inguinal lymph node area for a total of two injections, with phosphate-buffered saline (PBS) as a negative control immunization. In the adjuvant screen experiment, each mouse per group (*n* = 10) was immunized two times with M-M (50 μg) combined with different adjuvants as follows: BCG (50 μg, Founding Company, Shijiazhuang, China), Thymosin α1 (Tα1, 30 μg, DIAO, Chengdu, China), R848 (20 μg, Haoran Bio Technologies Co, Shanghai, China), A-type CpG 1585 (5′-GGGGTCAACGTTGAGGGGGG-3′, 50 μg, Sangon Biotech, Shanghai, China), B-type CpG 1826 (5′-tccatgacgttcctgacgtt-3′, 50μg, Sangon Biotech, Shanghai, China) (lowercase letters represent phosphorothioate linkage), and PBS as a negative control. In CpG 1826 dose screen experiment, six mice per group were immunized two times with CpG 1826 10, 30, 50 μg alone or in combination with M-M (50 μg). M-M (50 μg) combined with BCG (50 μg) was used as a positive control immunization. Studies were performed in accordance with the guidelines established by the Jilin University Institutional Animal Care and Use Committee (approved on 1 January 2016, Protocol No. 2015-34).

### 4.3. Tumor Protection in a Prophylactic Model

The effect of vaccination with different immunogenic adjuvants on tumor growth and immune activity was evaluated in a prophylactic model. The mice were randomly divided into seven groups of ten animals and were immunized as described above. One week after the final immunization, the tumor challenge was performed with an s.c. injection with 2 × 10^6^ B16-*MUC1* cells. To evaluate the dose effect of CpG 1826 on tumor growth and activity, the mice were randomly divided into eight groups of six mice. The mice were immunized as described above. One week after the final immunization, the tumor challenge was performed with an s.c. injection with 5 × 10^5^ B16-*MUC1* cells. Tumor size was determined by taking perpendicular measurements with calipers every 2–3 days, and the tumor volume (mm^3^) was calculated using the following formula: (*a* × *b*^2^)/2, where *b* is the smaller of the two measurements. At 24 days after tumor challenge, the mice were sacrificed.

In tumor challenge experiments, mice (*n* = 10) were immunized with PBS, M-M (50 μg), CpG 1826 (50 μg), or M-M + CpG 1826 subcutaneously for a total of two injections, a tumor challenge was performed with subcutaneous injection of 5 × 10^5^ B16-*MUC1* cells seven days after the final immunization. Mice were sacrificed at day 14 after tumor implantation.

### 4.4. ELISA for MUC1-Specific Immunoglobulin Subclasses

Seven days after the final immunization (or 14 days after tumor challenge), sera were isolated from the immunized mice, and the MUC1-specific antibodies were determined using enzyme-linked immunosorbant assay (ELISA). Briefly, 96-well plates were coated overnight at 4 °C with 10 µg/well of the MUC1 peptide. The MUC1 peptides of 30 amino acids used in the antibody, proliferation, and cytokine assays were constructed by Shanghai Ziyu Biotechnology. The wells were blocked with PBS containing 2% bovine serum albumin. For IgG, IgG1, and IgG2c antibody titer detection, the sera samples were diluted 1:500 and later incubated at 37 °C for 1.5 h. The plates were washed and incubated with horseradish peroxidase-labeled goat anti-mouse IgG, IgG1 and IgG2c (Sigma Chemical Co., St. Louis, MO, USA) for 1 h at 37 °C. The plates were then washed three times and were incubated with the substrate o-phenylenediamine dihydrochloride (OPD, Amresco, Solon, OH, USA) for 10 min, and 0.2 mM H_2_SO_4_ was added for terminate the reaction. The absorbance was measured using a microplate reader at a wavelength of 490 nm (BioTek Instruments, Inc. Winooski, VT, USA). The results are expressed as the average value ± standard deviation (SD).

### 4.5. MUC1 Specific Cell Proliferation and Th Activity Assay

Seven days after the final immunization (or 14 days after tumor challenge), splenic mononuclear cells were isolated using Ficoll (Solarbio, Beijing, China) from immunized mice and were cultured in IMDM containing 100 U/mL interleukin-2 with or without 20 μg/mL MUC1 synthetic peptide (Solarbio) at a density of 1 × 10^6^ cells/well at 37 °C in 5% CO_2_ for five days in 96-well Flat bottom plate. The culture supernatants were collected for a cytokine assay both by ELISA and the Quantibody^®^ array. The level of MUC1-specific IFN-γ production should be expressed as the level of cytokine secreted by splenic mononuclear cells stimulated by MUC1 peptide minus the amount of cytokines secreted by non-specific stimulated cells. Next, the WST-1 reagent (Dojindo Molecular Technologies, Tokyo, Japan) was added to each well for the MUC1-specific cell proliferation analysis. The IFN-γ production was assessed using an ELISA kit (eBioscience, Inc., San Diego, CA, USA) according to the manufacturer’s instructions.

The WST-1 reagent (Roche) was added to each well at a final concentration of 10% (*v*/*v*), and the plate was cultured in dark at 37 °C for 1 h. Next, the absorbance was measured using a microplate reader at a wavelength of 450 nm (BioTek Instruments, Inc. Winooski, VT, USA). The results are shown as the relative cell viability. The relative cell viability was calculated as A450 (MUC1-stimulated group)/A450 (control group).

### 4.6. Cytokine Assay by Quantibody^®^ Array

A Quantibody^®^ array was used to detect the changes of cytokines secreted by the MUC1-specific T cell. The cells were isolated and cultured as described above. Next, the cell supernatants were harvested and were analyzed for the presence of IFN-γ, IL-1b, IL-10, IL-13, IL-17A, IL-17F, IL-21, IL-22, IL-23, IL-28A, IL-4, IL-5, IL-6, MIP-3α, and TGF-β1. Briefly, a capture antibody was first bound to the glass surface. After the incubation with the sample, the target cytokine was trapped on the solid surface. Next, a second biotin-labeled detection antibody was added, which recognized a different epitope of the target cytokine. The cytokine-antibody-biotin complex was visualized by the addition of the streptavidin-conjugated Cy3 equivalent dye using a laser scanner.

### 4.7. MUC1-Specific CTL Cytotoxicity Assay

Cytotoxicity was measured using a lactate dehydrogenase (LDH) release assay (Promega Corporation, Madison, WI, USA). For MUC1-specific CTL cytotoxicity assay, splenic mononuclear cells from immunized mice were isolated using Ficoll and stimulated with MUC1 peptide for five days. The MUC1 recalled splenic mononuclear cells were used as effectors. The B16-*MUC1*, B16-neo, or LLC target cells were plated at a density of 1 × 10^4^ cells/well in U-bottomed 96-well plates at effector-to-target cell (E/T) ratios of 25:1, 12.5:1, and 6.25:1. Cells were incubated for 4h at 37 °C in an atmosphere of 5% CO_2_. Then the culture supernatant (50 μL/well) from each well was then transferred to a fresh 96-well plate. Fifty microliters of the reconstituted substrate mix was added to each well. After incubation at room temperature for 30 min, 50 μL of stop solution was added. The absorbance was measured at 490 nm using an ELISA reader. The percentage of cytotoxicity was calculated as follows: Cytotoxicity (%) = (effectors and target mixture − effectors spontaneous − target spontaneous)/(target maximum − target spontaneous) × 100%.

### 4.8. Analysis of DC Maturation by Flow Cytometry

The maturation of DC in vivo and in vitro was analyzed by flow cytometry. For the in vivo study, the mice were randomly divided into four groups of ten animals. The mice were subcutaneously immunized with PBS, CpG 1826, M-M in the presence of CpG 1826 or not, two times with a two-week interval. Seven days after the final immunization, the mice were sacrificed. The draining lymph nodes were made into a single-cell suspension with a 200 mesh filter. In vitro, the bone marrow-derived DCs (BMDCs) were isolated from the hind limb bones of the mice using a published protocol [41]. Briefly, 10^6^ cells obtained from the femur were seeded into a six-well plate with RPMI 1640 supplemented with 10% FCS, 20 ng/mL of IL-4 (PeproTech, London, UK), and 20 ng/mL GM-CSF (PeproTech). The medium was replaced on days 3 and 5. The cells were harvested on day 6 and divided into three groups by adding of different reagents: bank control, M-M (10 μg /mL), CpG 1826 (10 μg/mL), and M-M + CpG 1826. After the BMDCs were incubated with the different stimulation reagents for 48 h, the BMDCs in the different groups were collected and prepared to use.

For flow cytometry, the cells were stained for 30 min on ice with following fluorescence-labeled antibodies (1 μg/2 × 10^6^ cells) purchased from eBioscience: anti-mouse MHCI-FITC; anti-mouse MHCII-FITC; anti-mouse CD11c-APC; anti-mouse CD86-FITC; anti-mouse CD80-FITC and anti-mouse CD40-FITC. The cells were then washed twice with cold PBS and were analyzed on BD Accuri C6. The data analysis was performed using BD Accuri C6 software (BD biosciences, San Jose, CA, USA).

### 4.9. Analysis of the CD4^+^ T Cell Purity

Mice were immunized with PBS, M-M (50 μg), CpG 1826 (50 μg), or M-M + CpG 1826 (50 μg) subcutaneously for a total of two injections. The spleen samples were obtained from each group and the mononuclear cells were separated using Ficoll. CD4^+^ T cells were isolated from this single-cell suspension using the CD4^+^ T Cell Isolation Kit II, an LS column, and a MidiMACS™ separator (Miltenyi Biotec, Bergisch Gladbach, Germany). Non-CD4^+^ T cells are indirectly magnetically labeled by using a cocktail of biotin-conjugated antibodies (10 μL/10^7^ total cells) and Anti-Biotin MicroBeads (20 μL/10^7^ total cells). The harvested CD4^+^ T cells were incubated with a FITC-labeled anti-CD4 antibody to determine the purity by flow cytometry before they were used.

### 4.10. Activity Analysis of the CD4^+^ T Cells Co-Cultured with the BMDCs

BMDCs were isolated and cultured as previous described and were analyzed by flow cytometry before they were used. The cells were divided into four groups by the addition of different reagents as follows: PBS; M-M (10 mg/mL); CpG 1826 (10 mg/mL); and M-M + CpG 1826. The plates were incubated for 48 h. Next, the purified CD4^+^ T cells were cocultured with the BMDCs at a ratio of 50:1 in a 96-well and cultured for 48 h. Finally, the supernatants were collected for the cytokine assay. WST-1 was added to the wells for the cell proliferation assay. The levels of IFN-γ, IL-4 and IL-12p70 production in the supernatant were also detected by an ELISA kit (eBioscience). The experiment was replicated three times. The absorbance at 450 nm in each well was measured with an automated microtiter plate reader.

### 4.11. Survival of Mice in the Prophylactic and Therapeutic Model

In the prophylactic study, the mice were randomly divided into four groups of ten animals and were treated with the following agents: CpG 1826, M-M + CpG 1826, M-M + BCG, and PBS as a negative control. One week after the final immunization, a tumor challenge was performed with an s.c. injection with 5 × 10^5^ B16-*MUC1* cells.

In the therapeutic study, the mice were randomly divided into four groups of ten animals. For the tumor challenge, the mice were injected s.c. with 5 × 10^5^ B16-*MUC1* cells into the back near the neck. Seven days after the tumor challenge, the mice were treated with the following agents: CpG 1826, M-M + CpG 1826 or M-M + BCG, and PBS as a negative control. A total of two vaccine injections were spaced 14 days apart. Tumor size was determined by taking perpendicular measurements with calipers every 2–3 days. The tumor volumes were monitored over an 80-day post-tumor implantation period.

### 4.12. Statistical Analysis

Tumor growth curves were plotted based on tumor size until the first mouse died. One-way analysis of variance (ANOVA) was used to analyze the experimental data. A two-sided Student’s *t*-test was adopted to compare the mean values of individual treatments when the primary outcome was statistically significant. Survival was estimated by the Kaplan–Meier method and evaluated with a log-rank test. *p* < 0.05 was considered as statistically significant. All statistical analyses were performed with SPSS 18.0 software.

## Abbreviations

MBP	Maltose-Binding Protein
M-M	MUC1-MBP
CpG	CpG Oligodeoxynucleotides
TLR	Toll-like Receptor
B16-*MUC1*	*MUC1*-Overexpressed B16 Melanoma Cells
BCG	Bacillus Calmette-Guérin
APC	Antigen Presenting Cell
BMDCs	Bone Marrow Dendritic Cells
mDC	Marrow Derived DCs
pDC	Plasmacytoid Dendritic Cells
dLNs	Draining Lymph Nodes
SI	Stimulation Index
CTL	Cytotoxic T Lymphocytes
